# Salvage lenvatinib/everolimus combination therapy after immune checkpoint inhibitor and VEGFR tyrosine kinase inhibitor for metastatic renal cell carcinoma

**DOI:** 10.3389/fonc.2023.1231831

**Published:** 2023-07-27

**Authors:** Christopher Kwok, Adam Khorasanchi, Sarah P. Psutka, Megan Hinkley, Shawn Dason, Debasish Sundi, Yuanquan Yang, Yajing Yang, Claire Verschraegen, Evan E. Gross, Delaney Orcutt, Ming Yin

**Affiliations:** ^1^ Department of Pharmacy, The Ohio State University Comprehensive Cancer Center, Columbus, OH, United States; ^2^ Division of Medical Oncology, The Ohio State University Comprehensive Cancer Center, Columbus, OH, United States; ^3^ Department of Urology, University of Washington, Fred Hutchinson Cancer Center, Seattle, WA, United States; ^4^ Division of Urologic Oncology, The Ohio State University Comprehensive Cancer Center, Columbus, OH, United States; ^5^ The University of Washington School of Medicine, Seattle, WA, United States

**Keywords:** lenvatinib, everolimus, immune checkpoint inhibitor, VEGFR tyrosine kinase inhibitor, renal cell carcinoma, retrospective review

## Abstract

**Background:**

The optimal treatment for metastatic renal cell carcinoma (mRCC) patients who have progressed after both immune checkpoint inhibitor (ICI) and VEGFR tyrosine kinase inhibitor (TKI) remains uncertain. Lenvatinib and everolimus (LE) are frequently used in combination as salvage therapy because of their different antitumor mechanisms, but efficacy and toxicity data in this setting are lacking.

**Methods:**

We retrospectively reviewed charts from two academic centers for 71 adult mRCC patients who received LE after prior ICI and TKI exposure. We evaluated patient demographics, histology, International mRCC Database Consortium (IMDC) risk group, treatment history, and toxicity details. Outcomes of interest included objective response rate (ORR), time to treatment failure (TTF), overall survival (OS), ≥grade 3 toxicities, and schedule or dosage changes, which were evaluated using descriptive statistics, chi-square test, Cox proportional hazards model, and the Kaplan–Meier method.

**Results:**

The median age was 64 (range 31–84). Most patients had clear cell histology (84.5%) and had undergone nephrectomy (80.3%). IMDC risks were favorable (19.7%), intermediate (int) (66.2%), poor (11.3%), and unknown (2.8%). The average ORR was 26.8%, while the median TTF was 5.5 months (95% confidence interval [CI], 3.5–7.6) and the median OS was 9 months (95% CI, 7.6–12.9). Intermediate and poor IMDC risks were independently associated with a significantly worse TTF compared to favorable risk (hazard ratio (HR), 3.03, 95% CI, 1.18–7.79), as was ≥4L treatment *vs.* 2L/3L treatment (HR, 2.02, 95% CI, 1.08–3.8). Of the 71 patients, 57.7% had ≥grade 3 adverse events, 60% had treatment interruption, 44.3% had dose reduction, and 21% stopped treatment due to intolerance.

**Conclusions:**

LE therapy is feasible but has modest efficacies following ICI/TKI treatment. Patients with favorable risk or treated earlier may have a better treatment response. These observations need to be confirmed in prospective studies.

## Introduction

The treatment landscape for metastatic renal cell carcinoma (mRCC) is continuously evolving. The current first-line (1L) treatment approach includes the use of vascular endothelial growth factor receptor tyrosine kinase inhibitor (VEGFR-TKI)- and immune checkpoint inhibitor (ICI)-based combination therapies (1). Unfortunately, most mRCC patients will ultimately develop resistance to frontline therapy, and the optimal treatment for those who progress after both TKI and ICI remains uncertain.

The ideal salvage treatment should possess unique antitumor mechanisms when compared to TKI and ICI and demonstrate robust clinical efficacy with tolerable toxicities. Of all United States Food and Drug Administration (FDA)-approved agents for mRCC treatment, lenvatinib and everolimus (LE) combination therapy has emerged as a viable salvage regimen in this setting. Lenvatinib is a pleiotropic kinase inhibitor that targets VEGFR, fibroblast growth factor receptors (FGFRs), platelet-derived growth factor receptor (PDGFR), RET, and KIT (2). Everolimus is a mammalian target of rapamycin (mTOR) inhibitor, which targets the PI3K/AKT/mTOR pathway. In RCC, these pathways are often dysregulated and associated with poor prognosis (3). In 2016, the FDA approved LE after one prior anti-angiogenic therapy based on an open-label randomized phase II trial of 153 patients who were naïve to immunotherapy. These patients were randomly allocated to receive the combination of lenvatinib plus everolimus (n = 51), single-agent lenvatinib (n = 52), or single-agent everolimus (n = 50). The combination showed a superior median progression-free survival (mPFS; 14.6 *vs.* 5.5 months) and median overall survival (mOS; 25.5 *vs.* 15.4 months) and higher overall response rate (ORR; 43% *vs.* 6%) when compared to everolimus (4).

Today, LE is frequently used as a salvage treatment, but efficacy and toxicity data from the ICI era are limited to the initial second-line phase II trial and small retrospective, single-center studies. A 2020 case series of seven patients found improved PFS in mRCC patients treated with salvage LE (3–15 *vs.* 1.5 months) (5). Additionally, a single-center retrospective study published an ORR of 21.8%, an mPFS of 6.2 months, and a mOS of 12.1 months in 55 mRCC patients treated with lenvatinib with or without everolimus following ICI and TKI therapies (6). Lastly, another retrospective study assessed the efficacy of LE in 79 heavily pretreated mRCC patients in a community oncology setting and reported an ORR of 55.7%, with a median duration of response of 9.7 months and an mPFS of 6.1 months (7). Of these 79 patients, only 10 had prior TKI and ICI. Collectively, these studies suggest that LE may be efficacious as a salvage treatment for mRCC patients. Here, we retrospectively evaluated the efficacy and toxicity of LE as a salvage treatment in mRCC patients at two referral centers, previously treated with ICI and TKI therapies, the current standard of care for first-line treatment.

## Patients and methods

### Patient population

We retrospectively collected data between 05/01/2015 and 05/01/2021 from The Ohio State University Comprehensive Cancer Center (OSU) and the University of Washington (UW)—Fred Hutchinson Cancer Center (FHCC). The study was approved by the Institutional Review Board and Ethics Committee at both institutions, and patient consent was waived given the retrospective observational study design. We identified 71 patients with mRCC of any histology who were at least 18 years of age and who had received salvage LE therapy after prior exposure to both ICI-based systemic therapy (consisting of pembrolizumab, nivolumab, avelumab, or nivolumab-ipilimumab) and TKI therapy (including sunitinib, axitinib, pazopanib, lenvatinib, or cabozantinib). Patients were excluded if they received ICI/TKI therapy elsewhere, as those subjects’ clinicopathological data were not universally available. None had lenvatinib exposure prior to LE treatment.

### Data and outcomes

Clinical data including patient demographics, histologic subtype, International mRCC Database Consortium (IMDC) risk group, RCC treatment history, ICI/TKI regimens, and lines of therapy were recorded. IMDC risk was calculated at the time of first-line therapy. Measurements of ORR, time to treatment failure (TTF), OS, and records of safety/toxicity were used for the assessment of clinical outcomes. ORR was defined as the best response by complete response (CR) or partial response (PR), with treatment response evaluated by medical oncologists based on Response Evaluation Criteria in Solid Tumors (RECIST) 1.1 criteria. Patients without measurable disease were excluded from ORR analysis. TTF was defined as the time of LE therapy initiation to the date of radiographic disease progression, treatment discontinuation due to toxicity, patient preference, or death from any cause. OS was defined as the time of LE therapy initiation to the date of last follow-up or death from any cause. Safety and tolerability were determined by related descriptions in chart review and were assessed for grade ≥3 treatment-related adverse events (AEs) based on the Common Terminology Criteria for Adverse Events Version 5.0, and schedule changes or dosage modifications.

### Statistical analysis

Details regarding demographics, disease, treatment, and toxicity characteristics were summarized by descriptive statistics. A chi-square test was performed to compare ORR between groups. TTF and OS were estimated using the Kaplan–Meier method. Univariable and multivariable Cox proportional hazards ratio analyses were performed to assess TTF and OS outcomes. Multivariable analysis was performed by adjustment of all clinicopathological factors, including age, gender, LE lines of therapy, history of nephrectomy, histology, IMDC group, and prior ICI/TKI exposure method (sequential *vs.* combinational). All statistical procedures were performed using SAS 9.4 software (SAS, Inc.). A p-value <0.05 was considered statistically significant.

## Results

### Patient characteristics

A total of 71 patients with mRCC were included, including 42 from OSU and 29 from UW/FHCC. The median follow-up for survivors was 11.7 months (range 2.7 – 36.6; interquartile range [IQR] 6.6–14.1). The clinicopathological characteristics of the cohort are presented in **Table 1**. The median age was 64 years (range 31.3–84.3; IQR 57.7–70) for the entire cohort. The majority of the patients were male (80.3%, N = 57), and the most common histology was clear cell (84.5%, N = 60), with 11 (15.5%) patients having non-clear cell RCC, including papillary type (3), chromophobe type (2), and unclassified type (6).

**Table 1:** Patient characteristics.

Parameters	Total (%)
**Number**	71
**Median age (range)**	64 (31.3–84.3)
Sex	
Male	57 (80.3)
Female	14 (19.7)
Race	
White	64 (90.1)
Black	3
Other	4
Histology	
Clear cell	60 (84.5)
Papillary	3 (4.2)
Chromophobe	2 (2.8)
Other	6 (8.5)
IMDC risk group	
Favorable	14 (19.7)
Intermediate	47 (66.2)
Poor	8 (11.3)
Unknown	2 (2.8)
Nephrectomy	
Yes	57 (80.3)
No	14 (19.7)
Metastatic site	
Lymph node	20 (28.2)
Lung	35 (49.3)
Liver	8 (11.3)
Bone	25 (35.2)
CNS	1 (1.4)
Muscle	2 (2.8)
Local tumor bed	5 (7)
Other kidney	2 (2.8)
Others	11 (15.5)
Lenvatinib/everolimus line of therapy	
2	2 (2.8)
3	19 (26.8)
4	15 (21.2)
> 4	35 (49.3)
Prior regimen	
ICI/TKI combination ± Others*	3 (4.2)
ICI + ICI/TKI combination ± Others	10 (14.1)
TKI + ICI/TKI combination ± Others	4 (5.6)
TKI + ICI ± Others	42 (59.2)
TKI + ICI + ICI/TKI combination ± Others	12 (16.9)

IMDC, International mRCC Database Consortium; CNS, central nervous system; ICI, immune checkpoint inhibitor; TKI, tyrosine kinase inhibitor.

*Others include bevacizumab, IL2, interferon, and investigating agents.

Fourteen patients (19.7%) had favorable risk by IMDC criteria, 47 (66.2%) intermediate risk, and 8 (11.3%) poor risk. The majority of patients had undergone prior nephrectomy (80.3%, N = 57). The three most common metastatic sites were the lung (49.3%), bone (35.2%), and lymph node (28.2%).

**Table 1** shows the treatment history of this patient population. Patients received a median of 3 (range 1–10) therapy lines prior to LE receipt. LE combination therapy was used in 2, 19, 15, and 35 patients in the second-line (2L), third-line (3L), fourth-line (4L), and subsequent treatment (>4L) settings, respectively. The details of prior treatment exposure are also shown, which can be further grouped based on how ICI and TKI were administered previously. Forty-two patients (59.2%) received sequential TKI and ICI therapy (group: TKI + ICI ± Others), while the remaining patients received ICI/TKI combination therapy prior to LE treatment.

### Efficacy

As shown in **Table 2**, 57 out of the 71 patients had measurable responses for ORR analysis, including 15 PR, 28 stable disease (SD), and 14 progressive disease (PD). Overall, the ORR was 26.3%, which was not significantly different based on lines of therapy (2L/3L *vs.* ≥4L: 28.6% *vs.* 25%; p = 0.77). Prior ICI and TKI treatment methodology (sequential or combination) did not seem to impact the ORR of LE salvage therapy (sequential *vs.* combination: 22.2% *vs.* 28.6%; p = 0.59). We attempted to assess the efficacy of LE in non-clear RCC. Nine out of the 11 patients had evaluable responses. The ORR was 33% (3 PRs), which was not significantly different from the ORR in clear-cell RCC (25%, p = 0.6).

**Table 2:** ORR by line of therapy.

Line of therapy	Patient No.	CR	PR	SD	PD	ORR (%)
Second line	2	0	1	1	0	50
Third line	19	0	5	10	4	26.3
**Sub-total**	21	0	6	11	4	28.6
Fourth line	11	0	1	7	3	9
> Fourth line	25	0	8	10	7	32
**Sub-total**	36	0	9	17	10	25
**Total**	**57**	**0**	**15**	**28**	**14**	**26.3**

Response not measurable/available in 14 patients.

ORR, objective response rate; CR, complete response; PR, partial response; SD, stable disease; PD, progressive disease.

The bold values are meant to highlight the total number of patients with measurable responses for ORR analysis.

**Figure 1:**
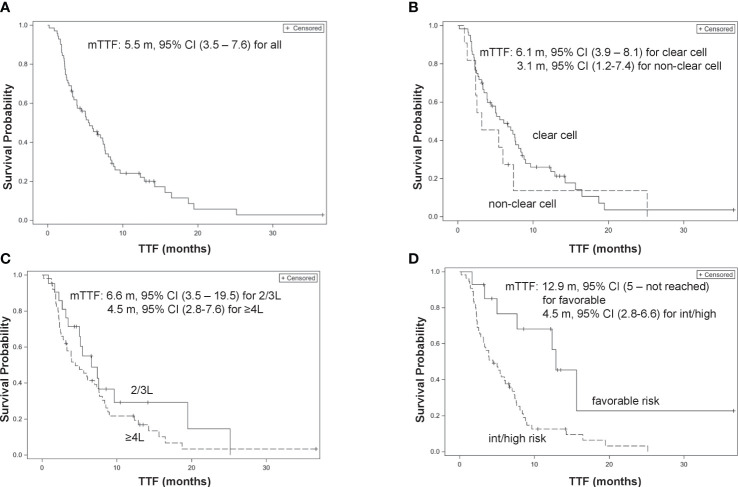
Kaplan–Meier curves of time-to-treatment failure (TTF). **(A)** Overall patients. **(B)** Clear cell type *vs.* non-clear cell type. **(C)** 2/3L treatment *vs.* **≥**4L treatment. **(D)** International mRCC Database Consortium (IMDC) favorable risk *vs.* intermediate/poor risk group.

All 71 patients were eligible for TTF analysis with a median TTF of 5.5 months (95% confidence interval [CI], 3.5–7.6 months). On univariable analyses, a history of nephrectomy was associated with a better TTF (hazard ratio (HR), 0.46, 95% CI, 0.25–0.85; p = 0.013), while intermediate and poor IMDC risks were associated with a significantly worse TTF (HR, 3.19, 95% CI, 1.44–7.1; p = 0.004). On multivariable regression analyses, intermediate and poor IMDC risks and ≥4L treatment were independently associated with a significantly worse TTF (adjusted HR [adjHR], 3.03, 95% CI, 1.18–7.79; p = 0.021 and adjHR, 2.02, 95% CI, 1.08–3.8; p = 0.03, respectively), while histology of non-clear cell type was associated with a borderline significantly worse TTF (adjHR, 2.06, 95% CI, 0.99–4.28; p = 0.052) (**Table 3**; **Figure 1**).

**Table 3:** Associations between clinicopathological factors and TTF.

	Univariable			Multivariable*		
	HR	95% CI	p	HR	95% CI	p
Age						
≤64	1			1		
>64	0.6	0.36–1.01	0.055	0.57	0.3–1.07	0.08
Gender						
F	1			1		
M	1.7	0.81–3.58	0.159	1.83	0.83–4.07	0.137
Histology						
Clear cell	1			1		
Others	1.38	0.69–2.76	0.369	2.06	0.99–4.28	0.052
Lines of therapy						
2/3L	1			1		
≥4L	1.46	0.81–2.64	0.206	2.02	1.08–3.8	0.03
Nephrectomy						
No	1			1		
Yes	0.46	0.25–0.85	0.013	0.6	0.29–1.25	0.172
IMDC						
Favorable	1			1		
Int/poor	3.19	1.44–7.1	0.004	3.03	1.18–7.79	0.021
Prior ICI/TKI						
Sequential	1			1		
Combinational	0.96	0.56–1.64	0.889	0.64	0.35–1.17	0.149

TTF, time to treatment failure; IMDC, International mRCC Database Consortium; ICI, immune checkpoint inhibitor; TKI, tyrosine kinase inhibitor.

*Adjusted by age, gender, LE lines of therapy, history of nephrectomy, histology, IMDC group, and prior ICI/TKI exposure method (sequential vs. combinational)

**Figure 2:**
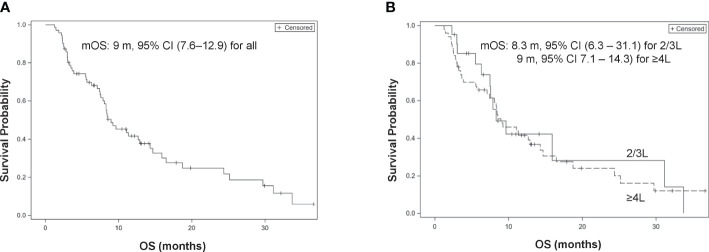
Kaplan–Meier curves of overall survival (OS). **(A)** Overall patients. **(B)** 2/3L treatment *vs.* **≥**4L treatment.

All patients were eligible for OS analysis with a median OS of 9 months (95% CI, 7.6–12.9). Among 71 patients, 50 died at the time of data cutoff. Late administration of LE (≥4L) was not associated with a worse OS compared with 2L and 3L treatments (HR, 1.11, 95% CI, 0.59–2.11; p = 0.74 and adjHR, 1.62, 95% CI, 0.81–3.24; p = 0.175, respectively) (**Figure 2**).

### Safety of salvage LE therapy

Of the 71 patients, 41 (58%) developed a total of 49 different AEs of grade 3 or higher related to LE. The most common ≥G3 AEs were others (33.8%), diarrhea/colitis (9.6%), and mucositis (7%) (**Table 4**). Approximately 60% of patients had treatment interruption, 44.3% had a dose reduction, and 21% stopped treatment due to intolerance. There were no treatment-associated deaths.

**Table 4:** Incidence of grade ≥3 AE (%).

Total (N = 71)	49
Liver toxicity	2 (2.8)
Diarrhea/colitis	7 (9.6)
Skin toxicity	4 (5.6)
Kidney damage	3 (4.2)
Lung	3 (4.2)
Mucositis	5 (7)
Musculoskeletal	1 (1.4)
Other	24 (33.8)

AE, adverse event.

## Discussion

This study is the largest case series to date examining the utility of LE therapy in patients with mRCC who had prior exposure to both ICI and TKI treatment. Our data suggest that salvage LE treatment is tolerable and feasibly administered with modest antitumor activity and moderate rates of significant treatment-associated adverse events in just over half of the patients. Patients with favorable risk, clear-cell histology, or early salvage may have a better treatment response. The retrospective data from two major United States academic centers provide real-world experiences and may warrant further investigations in larger clinical trials.

The treatment of mRCC has evolved from single-agent therapy to dual-agent therapy. KEYNOTE-426 (pembrolizumab-axitinib), Javelin Renal 101 (avelumab-axitinib), CheckMate-9ER (nivolumab-cabozantinib), and CLEAR trial (pembrolizumab-lenvatinib) were pivotal trials that established the superiority of the combination of PD-1/PD-L1 inhibitors plus a second-generation TKI over sunitinib monotherapy in all mRCC patients (8–11), with ORRs of 52.5%–71% and mPFS of 13.8–23.9 months in the 1L setting. The CheckMate-214 trial (nivolumab–ipilimumab) established the superiority of the combination of a PD-1 inhibitor plus a CTLA-4 inhibitor over sunitinib monotherapy in intermediate/poor-risk mRCC patients, with an ORR of 42% and an mPFS of 11.6 months in the 1L setting (12). Patients recurring after nivolumab–ipilimumab are usually treated with TKI-based therapy. As a result, almost all mRCC patients will have exposure to both ICI and TKI treatment within the first- or second-line settings. After disease progression to both ICI and TKI, the optimal treatment approach has not been defined. Rotating to a different TKI or using nivolumab-ipilimumab can be considered if not previously administered. However, a low response rate is observed, likely due to cross-resistance (13, 14). Identification of a regimen that retains significant antitumor activity after ICI and TKI is of high interest in clinical practice.

Lenvatinib is a multitarget tyrosine kinase inhibitor, which has not been approved for mRCC treatment as a monotherapy. Everolimus is an mTOR inhibitor that was approved after TKI treatment (sunitinib or sorafenib) in 2009 based on the RECORD-1 trial, which showed improved PFS (mPFS, 4.0 *vs.* 1.9 months), compared to placebo (15). The combination of lenvatinib and everolimus was approved in 2016 for the treatment of mRCC following one prior TKI based on a phase II trial. LE compared to sunitinib treatment in the 1L setting yields a better ORR (53.5% *vs.* 36.1%) and mPFS (14.7 *vs.* 9.2 months) (11). These observations seem to suggest that LE is superior to either TKI or everolimus monotherapy and can still be effective after prior TKI resistance. Since LE treatment incorporates everolimus, a drug with a different mechanism of action, it could be a good candidate therapy for mRCC patients with prior exposure to both ICI and TKI, but data are limited.

Our study provides important evidence to support the utilization of salvage LE therapy in select mRCC patients with a moderate but certain efficacy after ICI and TKI exposure. A reliable ORR of approximately 25% was observed, regardless of prior therapies or tumor histologies (<4L *vs.* ≥ 4L treatments; sequential *vs.* ICI/TKI combination; clear-cell *vs.* non-clear cell). LE therapy lasted for a median TTF of 5.5 months, substantially shorter than the mPFS of 14.6 months seen after one prior TKI, likely due to differences in patient population (4). Although TTF is not equivalent to PFS, the number is not significantly different since the majority of patients in our study continued LE treatment despite toxicities. Additionally, our efficacy data are in alignment with the 21.8% ORR and the 6.1 months median PFS reported in the retrospective study of 55 patients (6). With the size of our cohort, we were able to determine several patient characteristics that might correlate with better outcomes, including clear-cell histology, favorable IMDC risk, and early use of salvage LE therapy. Those results are not unexpected and are supported by findings from the literature. LE yields a 9.2-month mPFS in treatment-naïve non-clear cell mRCC (16), which appears lower than the 14.7 months mPFS in clear cell mRCC (11). Metastatic RCC of favorable IMDC risk tends to express pro-angiogenetic molecular features more robustly compared to intermediate or poor IMDC risks and hence may obtain more benefits from TKI-based treatment (17). The efficacy of systemic treatment decreases with increasing lines of therapy, which usually correlates with the accumulation of treatment-related resistance by tumor genetic or epigenetic alterations (13). However, how the addition of mTOR signaling inhibition impacts mRCC patients of different subgroups has not been elucidated.

Finally, although our study did not support a significant OS benefit by early LE administration than late LE administration, the prolonged TTF is still meaningful. In our view, a prolonged TTF but not OS suggests that many other factors such as patient characteristics, subsequent treatments, or tumor evolution may contribute to the overall hazard of death, which were not well addressed in this retrospective study. However, delaying the progression of metastatic disease is a worthy goal. Many randomized clinical trials of new agents in metastatic solid tumors have gained drug approval by showing improvement in PFS, without a corresponding increase in OS (18). Additionally, our data showed that salvage LE is safe and tolerable. Of the patients, 57.7% experienced ≥G3 AEs during treatment, which was lower than 83.1% observed in the 1L setting and 71% observed in the 2L setting. This could be due to patient selection effects since some patients might not be considered for LE salvage if they had significant toxicities from previous TKI treatment. Additionally, most patients in practice start treatment at lower doses than those used in clinical trials. Excluding the “Other” group, diarrhea/colitis was found to be the most common AE, which is consistent with the side effect profile reported previously (4, 11). No new safety signals were detected. Only 21% of patients stopped treatment due to toxicity or intolerance, whereas other studies have reported a discontinuation rate of 24%–33% (4, 7). The majority of the patients discontinued treatment due to progressive disease.

Our study has several limitations. First, it is limited by the inherent biases related to the retrospective study design and modest sample size as well as heterogeneity in prior lines of therapy and histology. Additional confounding factors such as patient comorbidity may not have been collected and hence could not be adjusted for in the multivariable analyses. The objective response and toxicity assessments were locally reviewed by the treating providers. Additionally, patients who had 1L/2L treatments at other centers were excluded, which could have introduced potential selection biases into this study. Second, the modest sample size did not allow us to further evaluate the data stratified by different ICI/TKI exposures (e.g., nivolumab/cabozantinib and pembrolizumab/axitinib) or to assess TTF in non-clear cell mRCC. Our sample size should also be considered relatively small, and a pooled analysis from all published articles would be ideal to address the clinical question. Third, our study could evaluate only those correlations between clinicopathological features and treatment outcomes. Further analyses based on serum biomarkers or tumor genomic profiles may reveal potential molecular predictors associated with response (19); however, we lack such data.

Nevertheless, our data provide real-world evidence that salvage LE therapy is feasibly delivered with modest efficacy following ICI/TKI treatment. Patients with favorable risk, clear-cell histology, or early salvage may demonstrate a better treatment response to this combination approach. The combination of LE was tolerable as a salvage regimen, but close toxicity monitoring is important given that 58% of patients developed ≥grade 3 adverse events on treatment. These observations need to be confirmed in prospective studies, such as NCT 05012371, which compared LE to cabozantinib for second- or third-line mRCC treatment.

## Data availability statement

The raw data supporting the conclusions of this article will be made available by the authors, without undue reservation.

## Ethics statement

Ethical review and approval was not required for the study on human participants in accordance with the local legislation and institutional requirements. Written informed consent for participation was not required for this study in accordance with the national legislation and the institutional requirements.

## Author contributions

MY and SP: conception and design. MY, CK, AK, and SP: acquisition of the data. MY and SP: analysis and interpretation of the data. MY, CK, AK, and SP: drafting of the manuscript. All authors: critical revision of the manuscript. MY: statistical analysis. MY: supervision. All authors contributed to the article and approved the submitted version.
